# Comparative evaluation of a Technicon SMAC2/RA1000 System with an American Monitor Parallel during normal service work

**DOI:** 10.1155/S1463924686000408

**Published:** 1986

**Authors:** A. J. Little, D. P. Jones, D. Thompson, Sherry Faye, M. Cumberbatch

**Affiliations:** 1 Department of Chemical Pathology St. James's University Hospital Leeds LS9 7TF UK; 2 Department of Chemical Pathology Leeds General Infirmary Leeds LS1 3EX UK; 3 Pathology Department Riyadh Armed Forces Hospital PO Box 7897 Riyadh 11159 Saudi Arabia


Journal of Automatic Chemistry/*Journal of Clinical Laboratory Automation*, Vol. 8, No. 4 (October–December 1986), pp. 207–210

### Comparative evaluation of a Technicon SMAC2/RA1000 System with an American Monitor Parallel during normal service work

**A. J. Little, D. P. Jones**
*Department of Chemical Pathology, St. James's University Hospital, Leeds LS9 7TF, UK*

**D. Thompson, Sherry Faye and M. Cumberbatch***
*Department of Chemical Pathology, Leeds General Infirmary, Leeds LS1 3EX, UK*

### Introduction

To meet the requirements of an increasing biochemical work-load, manufacturers have developed large and expensive computer-controlled analytical systems. Therefore the choice of main analyser for a clinical biochemistry department is a major decision with long-term financial implications. Advice is usually sought from current users, but this is often more anecdotal than factual and certainly not of a comparative nature. For many large departments, the choice is between two systems employing different analytical concepts. These are the Parallel (American Monitor, UK) and a combination of the SMAC2 and RA1000 analysers (Technicon, UK).

The Parallel is a recently introduced 30-channel, high-capacity, discretionary analyser, whilst the SMAC2 is an established 12–23 channel continuous-flow non-discretionary analyser which can only be made selective in terms of reporting. The RA1000 is a bench-top discretionary analyser operating like the Parallel but with much less capacity. The SMAC2 and RA1000 together form a system of similar capability to the Parallel. With both a Parallel and a SMAC2/RA1000 combination installed in the authors' separate departments in Leeds, a study has been undertaken to provide comparative data which should aid potential purchasers.

A SMAC2 was installed in the Chemical Pathology Department of the Leeds General Infirmary (LGI) in September 1982, followed by an RA1000 in October 1983. A Parallel was installed in the Chemical Pathology Department of St. James's University Hospital in January 1984. Both laboratories have a similar annual work-load and serve similar populations. The 15-week period of the evaluation took place during October to December 1984 against a background of normal routine service work. The analytical performance, reliability and running costs of both systems were investigated over this period.

* *Present address: Pathology Department, Riyadh Armed Forces Hospital, PO Box 7897, Riyadh 11159, Saudi Arabia.*

### Methods

All methodologies used were as recommended by the manufacturers. For the assessment of analytical performance, sufficient quantities of unassayed Gibcotrol (Gibco Diagnostics) freeze-dried control material at three nominal levels (Low: lot No. 194; Normal: lot No. 235; High: lot No. 191) were purchased. Wellcomtrol (Wellcome Reagents Ltd) SMAC High (lot No. K7519) was used on the Parallel for an elevated alkaline phosphatase level because of the analytical unsuitability of the Gibcotrol High material.

On one day a week, over a period of 15 weeks, each laboratory reconstituted one bottle of each material. The contents of the bottles were analysed randomly 10 times throughout the day for the commonly requested tests (see table 1). On each subsequent week the day was advanced by one in order that each of the five working days were covered on three occasions. For tests less frequently assayed, two aliquots of each material were measured during one analytical run per week (see table 2).

For the investigation of reliability, a detailed log of the daily working parameters, electromechanical and other failures were recorded throughout the whole three-month period. The relative running costs of the two systems were calculated for the whole of 1984, including staff, consumables and maintenance contracts.

### Results

### *Analytical performance*

The values obtained on the quality-control materials were analysed using the Statistics Package for Social Sciences (SPSS). Analysis of variance on the data showed that significant imprecision was between, rather than within, analytical runs. For each test, the overall mean, standard deviation and coefficient of variation is given in tables 1 and 2. Results are shown only for those tests common to both systems and where a test level was close to the detection limit of the assay, the results have been excluded from the tables. The data were reanalysed after removal of possible outliers using Healy's procedure [1] and the recalculated coefficients of variation are shown in brackets in tables 1 and 2.

### *Costs*

Details of capital costs can be found in a comprehensive review of large analysers based on manufacturers' information [2]. An estimate of the 1984 running costs for

*Table 1. Analysis of 150 measurements made on 15 days over a period of 15 weeks.*

| | | SMAC2 | | | Parallel | | |
|---|---|---|---|---|---|---|---|
| Test | | Mean | SD | CV | Mean | SD | CV |
| Sodium mmol/l | L | 123·2 | 1·16 | 0·9 (0·7) | 124·5 | 1·99 | 1·6 (1·4) |
| | N | 143·3 | 0·91 | 0·6 (0·6) | 144·3 | 1·99 | 1·4 (1·2) |
| | H | 150·6 | 1·20 | 0·8 (0·8) | 151·0 | 2·07 | 1·4 (1·3) |
| Potassium mmol/l | L | 2·24 | 0·082 | 3·7 (3·3) | 2·09 | 0·050 | 2 4 (1·5) |
| | N | 4·08 | 0·116 | 2·8 (2·9) | 4·23 | 0·167 | 4·0 (1·5) |
| | H | 6·52 | 0·181 | 2·8 (2·6) | 6·68 | 0·100 | 1·5 (1·3) |
| Chloride mmol/l | L | 85·9 | 0·97 | 1·1 (1·0) | 83·6 | 2·37 | 2·8 (2·0) |
| | N | 102·0 | 0·92 | 0·9 (0·7) | 104·3 | 2·55 | 2·4 (1·8) |
| | H | 106·3 | 1·04 | 1·0 (0·9) | 110·6 | 2·84 | 2·6 (1·9) |
| Bicarbonate mmol/l | L | 11·7 | 0·60 | 5·1 (4·4) | 11·0 | 1·74 | 15·8 (10·3) |
| | N | 24·8 | 0·79 | 3·2 (2·9) | 23·4 | 1·79 | 7·7 (4·7) |
| | H | 36·6 | 1·07 | 2·9 (2·9) | 35·6 | 3·46 | 9·7 (7·5) |
| Urea mmol/l | L | 1·75 | 0·16 | 9·1 (7·7) | 2·24 | 0·33 | 14·7 (13·2) |
| | N | 5·27 | 0·14 | 2·7 (2·3) | 6·05 | 0·36 | 6·0 (5·9) |
| | H | 26·77 | 0·44 | 1·6 (1·7) | 26·01 | 0·91 | 3·5 (2·3) |
| Creatinine umol/l | L | 111·8 | 3·03 | 2·7 (2·7) | 111·9 | 10·83 | 9·7 (8·2) |
| | N | 105·2 | 3·11 | 3·0 (2·7) | 100·0 | 14·21 | 14·2 (9·0) |
| | H | 611·5 | 8·96 | 1·5 (1·4) | 576·9 | 15·20 | 2·6 (2·4) |
| Total protein g/l | L | 41·7 | 0·95 | 2·3* (2·3)* | 41·1 | 1·05 | 2·6* (2·0)* |
| | N | 75·0 | 1·00 | 1·3 (1·2) | 71·0 | 1·91 | 2·7 (1·8) |
| | H | 90·7 | 1·34 | 1·5 (1·4)* | 80·6 | 3·75 | 4·6 (1·6)* |
| Albumin g/l | L | 21·0 | 0·44 | 2·1 (0·7) | 20·8 | 1·60 | 7·7 (5·1) |
| | N | 39·5 | 0·67 | 1·7 (1·4) | 39·3 | 1·41 | 3·6 (3·3) |
| | H | 46·0 | 0·68 | 1·5 (3·8) | 44·3 | 1·36 | 3·1 (3·0) |
| Bilirubin umol/l | N | 9·1 | 0·88 | 9·6 (7·1) | 10·2 | 2·21 | 21·6 (17·3) |
| | H | 168·0 | 6·65 | 4·0* (4·1)* | 166·4 | 6·72 | 4·0* (3·7)* |
| Alk. Phos. K.A.U/dl | N | 6·55 | 0·230 | 3·5 (3·6) | 4·29 | 1·890 | 44·8 (5·3) |
| | H | 20·74 | 0·660 | 3·2* (2·7)* | 30·15 | 1·089 | 3·6* (3·2)* |
| Calcium mmol/l | L | 1·661 | 0·026 | 1·6 (1·4) | 1·755 | 0·043 | 2·4 (2·2) |
| | N | 2·516 | 0·030 | 1·2 (1·1) | 2·485 | 0·041 | 1·6 (1·5) |
| | H | 3·422 | 0·046 | 1·3 (1·3) | 3·255 | 0·094 | 2·9 (1·8) |
| Phosphate mmol/l | L | 0·856 | 0·022 | 2·6 (2·3) | 0·916 | 0·035 | 3·8 (1·7) |
| | N | 1·128 | 0·025 | 2·2* (2·0) | 1·107 | 0·021 | 1·9* (1·4) |
| | H | 2·832 | 0·111 | 3·9* (3·4) | 2·937 | 0·101 | 3·4* (1·5) |

L – Gibco Low N – Gibco Normal H – Gibco High.
Figures in brackets are CV's after exclusion of outliers.
* Imprecisions of the analysers not significantly different ($P > 0·05$).

both systems is shown in table 3. Staffing costs are based on 1·2 times the mid-point of the appropriate salary scale. In the two laboratories similar functions are performed by different grades of staff. At St James's, demographic and test scheduling are performed by MLSOs, in contrast to clerical staff at the LGI. Furthermore, all samples for the Parallel are separated by the Parallel staff, whilst at the LGI samples for the SMAC2/RA1000 are processed by reception staff. These procedural differences have been allowed for by calculating the number of clerical and reception staff at the LGI employed on direct SMAC2/RA1000 work on a *pro rata* basis.

### *Reliability*

Data collected throughout the three-month period on the work-load and reliability are given in table 4.

### Discussion

The Parallel was designed to operate in a completely selective discretionary mode. However, at St. James's Hospital, because of a reluctance by clinical staff to request tests individually, three main profiles for urea and electrolytes, liver function and bone were offered, whilst remaining tests were measured individually. The order in which the samples were analysed was found to be important because, prior to any test analysis, appropriate reagent lines were purged if not immediately preceded by another analysis for the same test. This meant that the reagent consumption was much higher than expected and necessitated the grouping of the expensive tests where possible: either within the analytical run or on a less frequent batch basis. In particular, the high cost of the bicarbonate reagent discourages the dispersion of non-

*Table 2. Analysis of 30 measurements made on 15 days over a period of 15 weeks.*

| Test | | RA1000 mean | SD | CV | Parallel Mean | SD | CV |
|---|---|---|---|---|---|---|---|
| ALT IU/l | L | 14·2 | 2·4 | 17·0 (15·0) | 8·9 | 4·9 | 54·6 (38·0) |
| | N | 23·9 | 1·7 | 6·9 (6·7) | 16·5 | 4·1 | 24·8 (23·9) |
| | H | 159·3 | 9·5 | 10·5 (3·3) | 129·3 | 6·8 | 5·3 (5·1) |
| Uric acid umol/l | L | 0·143 | 0·007 | 4·9 (5·0) | 0·103 | 0·018 | 17·5 (15·7) |
| | H | 0·345 | 0·016 | 4·6* (2·6) | 0·287 | 0·012 | 4·2* (3·9) |
| | H | 0·569 | 0·022 | 3·9* (3·2)* | 0·529 | 0·018 | 3·4* (3·2)* |
| Triglyceride mmol/l | N | 0·29 | 0·19 | 63·7 (11·6) | 0·32 | 0·12 | 35·5 (31·1) |
| | H | 0·52 | 0·28 | 53·8 (5·4) | 0·45 | 0·12 | 26·9 (26·1) |
| Cholesterol mmol/l | L | 2·28 | 0·05 | 2·2 (2·0) | 2·18 | 0·36 | 16·3 (13·8) |
| | N | 4·13 | 0·09 | 2·1 (2·0) | 3·65 | 0·54 | 14·7 (11·3) |
| | H | 8·73 | 0·16 | 1·9 (1·8) | 7·43 | 1·19 | 18·2 (12·7) |
| Creatine kinase IU/l | L | 17·0 | 1·0 | 6·2 (6·2) | 24·5 | 11·5 | 47·2 (47·1) |
| | N | 35·4 | 3·4 | 9·5 (8·3) | 78·3 | 17·4 | 22·3 (20·9) |
| | H | 191·4 | 8·8 | 4·6 (4·6)* | 439·4 | 27·1 | 6·2 (5·2)* |

L– Gibco Low N – Gibco Normal H – Gibco High.
Figures in brackets are CVs after exclusion of outliers.
* Imprecisions of the analysers not significantly different ($P > 0{\cdot}05$).

*Table 3. Annual running costs.*

| Item | SMAC2/RA1000 | Parallel |
|---|---|---|
| Maintenance | £25 305 | £20 300 |
| Staffing | £46 254 | £42 496 |
| Dry consumables | £15 522 | £4 002 |
| Reagents | £9230 | £27 921 |
| Calibrators and controls | £5663 | £3709 |
| Total requests | 99 124 | 80 636 |
| Total tests | 1 122 192 | 582 960 |
| Total cost (−staff) | £55 720 | £55 932 |
| Total cost (+staff) | £101 974 | £98 428 |

*Table 4. Analyser work-load and reliability data.*

| | SMAC2 Mean | SMAC2 Observed range | Parallel Mean | Parallel Observed range |
|---|---|---|---|---|
| Time 1st result (24 h) | 10·24 | 9·55–11·30 | 12·57 | 11·00–16·10 |
| Time last result (24 h) | 15·56 | 14·55–17·35 | 16·55 | 15·30–20·00 |
| Total cups sampled | 601 | 415–779 | 471 | 296–665 |
| Total Q.C. cups | 50 | 35–62 | 78 | 30–197 |
| Patient samples | 473 | 343–616 | 363 | 176–590 |
| Repeat analyses | 62 | 30–192 | 26 | 0–140 |
| Dilutions | 16 | 6–35 | 1 | 0–6 |
| % Samples re-run | 16·8 | 9·4–56·5 | 7·7 | 0–57 |
| Routine maintenance per week (minutes) | 36 | 20–90 | 76 | 60–120 |
| Extra maintenance per week (minutes) | 11 | 0–30 | 99 | 45–189 |
| Downtime per week (minutes) | 47 | 0–220 | 117 | 15–420 |

electrolyte samples between those requiring bicarbonate. The Parallel was therefore operated in a mode not originally envisaged. At the LGI, a 12-channel SMAC profile (see table 1) was used for the analysis of the majority of samples. The other tests (see table 2) were measured on the RA1000 in batches.

*Precision*

During the study period both the SMAC2 and RA1000 performed better than the Parallel and the latter's imprecision did not greatly improve on exclusion of possible outliers. These findings for the SMAC2 are in accordance with data published in the Wellcome Quality Assurance Scheme end-of-term report (October 1985), which shows that the SMAC2 analyser group have lower imprecisions than the Parallel group for corresponding

*Table 5. Wellcome quality control scheme six-month imprecision values for the St. James's Hospital Parallel. (Six pairs.)*

| Test | Units | Imprecisions: Period May 85–October 85 | Imprecisions: Period November 84–March 85 |
|---|---|---|---|
| Albumin | g/l | 1·19 | 1·08 |
| Bicarbonate | mmol/l | 0·99 | 1·22 |
| Bilirubin | umol/l | 1·03 | 3·77 |
| Calcium | mmol/l | 0·046 | 0·093 |
| Chloride | mmol/l | 2·08 | 2·22 |
| Cholesterol | mmol/l | 0·136 | 0·297 |
| Creatinine | umol/l | 13·7 | 7·4 |
| Phosphate | mmol/l | 0·035 | 0·045 |
| Potassium | mmol/l | 0·055 | 0·057 |
| Protein | mmol/l | 1·12 | 1·91 |
| Sodium | mmol/l | 2·20 | 1·24 |
| Triglyceride | mmol/l | 0·153 | 0·182 |
| Urea | mmol/l | 0·36 | 0·71 |
| Uric acid | umol/l | 0·014 | 0·024 |
| ALT | IU/l | 2·05 | 3·86 |
| CK | IU/l | 15·4 | 52·0 |

channels. However, the report shows the RA1000 and Parallel groups to be more comparable, which is not reflected in our data. Since the period of the comparison the performance of the St. James's Parallel has improved, reflected by external quality assurance scheme returns (table 5).

*Reliability*

There were considerable problems with the mechanical reliability of the Parallel during the period of evaluation, the instrument being totally unusable on two full days. The problems were due to a computer failure necessitating a replacement computer being installed overnight by American Monitor, and, secondly, an electromechanical failure in the vial wash system. A further major contribution to the excessive downtime was the occasional halting of the Parallel during analysis due to a communications failure between the two system computers (this has been rectified by American Monitor). The downtime of the SMAC2 was predominantly due to replacing membranes during a run. The RA1000 downtime was approximately 15 min per week and was contributed to by lamp and sample probe problems.

*Costings*

In this study, during which little discretionary requesting was exercised or encouraged in either hospital, the total cost of running the two systems was very similar. Because of the considerable difference in the way the two hospitals handle similar work-loads, comparison of overall cost of service is more appropriate. Theoretically it might seem to be possible to reduce the overall cost at St. James's Hospital by encouraging a higher degree of selectivity in requests but as previously stated, this would be countered by increased reagent costs.

### Summary

Since our evaluation, there have been continuing in-the-field developments of the Parallel, in both hardware and software, which have improved the general performance of the instrument. Performance is also associated with operator familiarity with the instrument and the running-in time of a new instrument must not be underestimated. However, from the present study it is concluded that, overall, the SMAC2/RA1000 combination is more reliable and less imprecise than the Parallel.

### References

1. Healy, M. J. R., *Clinical Chemistry*, **25** (1979), 675.
2. Fyffe, J. A., *Communications in Laboratory Medicine*, **1** (1985), 118.

### SCIENTIFIC COMPUTING AND AUTOMATION

*13 to 15 May 1987: Amsterdam, The Netherlands.*

Europe's first conference specifically for computing and automation in the sciences will be held in Amsterdam in May 1987. Established as the European counterpart of its successful sister meeting in the USA, Scientific Computing and Automation (Europe) – SCAEUROPE – will be supported by a number of European societies, among them the Royal Society of Chemistry, UK, and the Royal Dutch Chemical Society. A full scientific programme, set up by a scientific board under the chairmanship of Professor D. L. Massart of the Vrije Universiteit, Brussels, will form the backbone of the meeting.

A number of short courses will be run in conjunction with the conference and a full exhibition of products and services, with special sessions on new developments, will also be included. It is intended that participants be provided with suitable computer facilities for 'hands-on' interface with exhibitors and other participants.

SCA Europe will devote both its inter-disciplinary and specialist parallel sessions to the latest advances in computing and automation in the scientific environment, including:

Chemistry (pharmaceutical, analytical, environmental, synthesis).
The life sciences (clinical, microbiological.
Engineering & technology (biotechnology, chemical technology).

Within the framework of these specialist fields, members of the international scientific community will be able to share and build upon their experiences in

Computing: off-the-shelf software, databases, image analysis, computer-aided learning (CAL), pattern recognition, computer-assisted organic synthesis, molecular modelling, computational chemistry, chemometrics, etc.
Automation: LIMS, networking, data acquisition, sensors and microsensors, process control, impact of new hardware on the laboratory, etc.
Robotics – laboratory products. Artificial intelligence and expert systems.

*Details from Scientific Computing and Automation (Europe), c/o Reunion International BV, Tholenseweg 3, 1/81 KD Amstelveen, The Netherlands. Tel.: 020 43 25 45.*

